# Supplementary material for: Synthesis and Identification of Epoxy Derivatives of 5-Methylhexahydroisoindole-1,3-dione and Biological Evaluation
Source: Molecules. 2021 Mar 30;26(7):1923. doi: 10.3390/molecules26071923 (PMC8037305; doi:10.3390/molecules26071923)
Supplement: Supplementary file 1 [file molecules-26-01923-s001.pdf]

SUPPLEMENTARY MATERIAL

Synthesis and Identification of Epoxy Derivatives of 5-Methylhexahydroisoindole-1,3-dione and Biological Evaluation

Kariny B. A. Torrent and Elson S. Alvarenga *

Department of Chemistry, Universidade Federal de Viçosa, Viçosa, 36570-900 MG, Brazil; karinybragatto@gmail.com
* Correspondence: elson@ufv.br

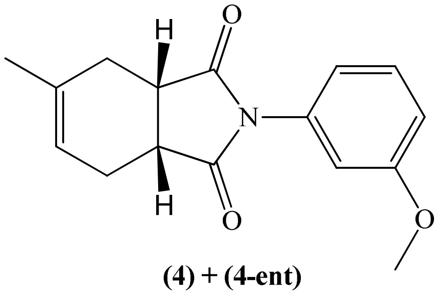

(4) + (4-ent)

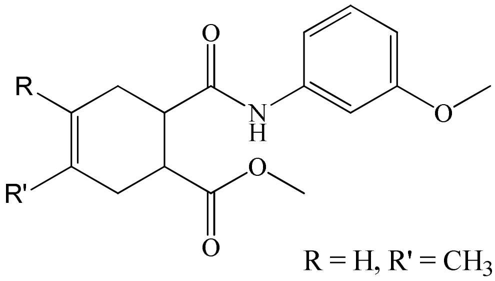

$R = H, R' = CH_3$

(3) +

$R = CH_3, R' = H$

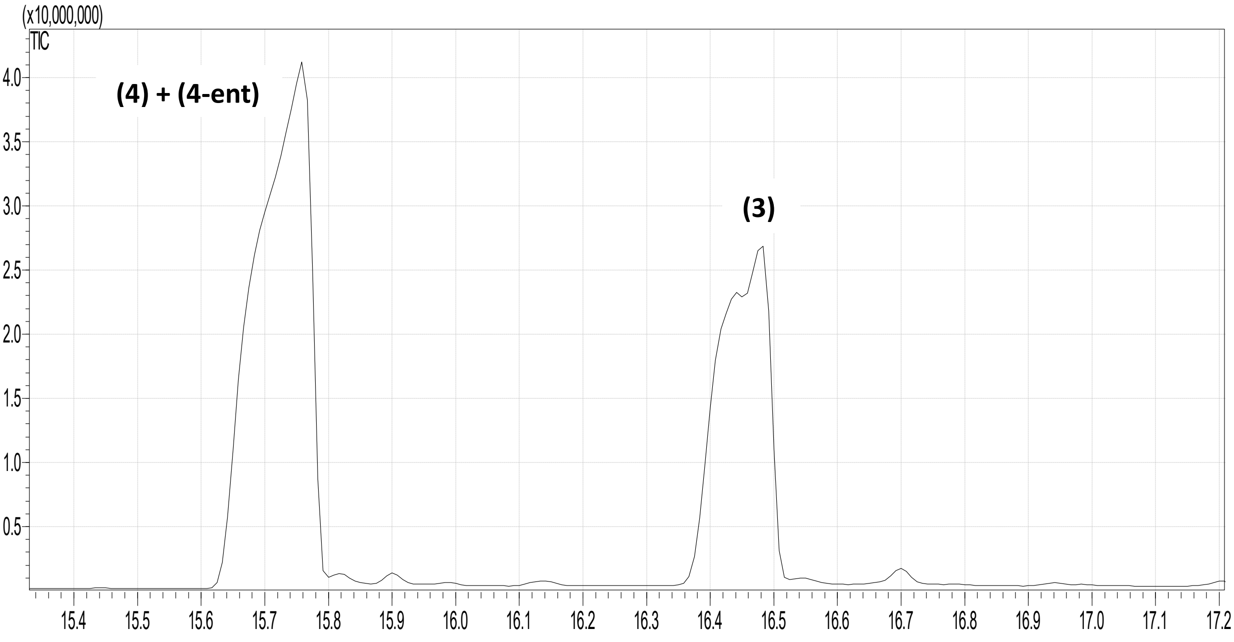

Figure S1 - Chromatogram of the mixture of tetrahydrophthalimide **(4 + 4-ent)** and ester **(3)**.

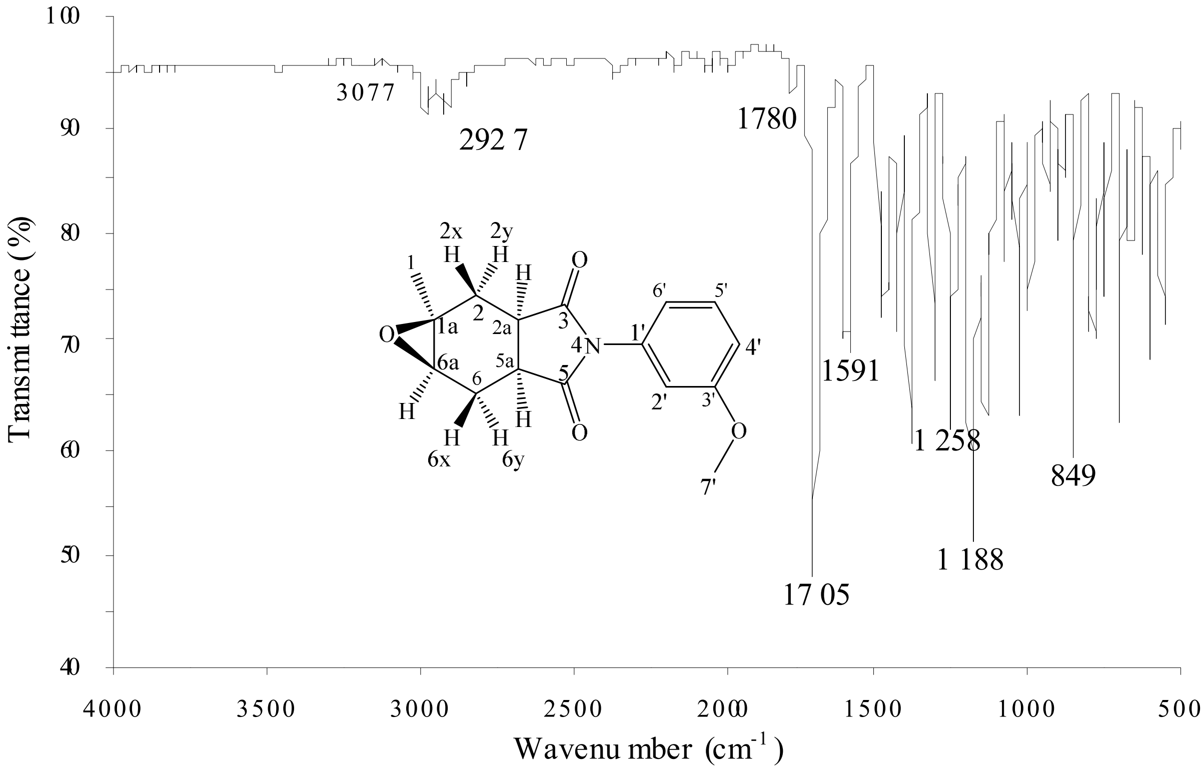

Figure S2: IR spectrum of compound **5b**.

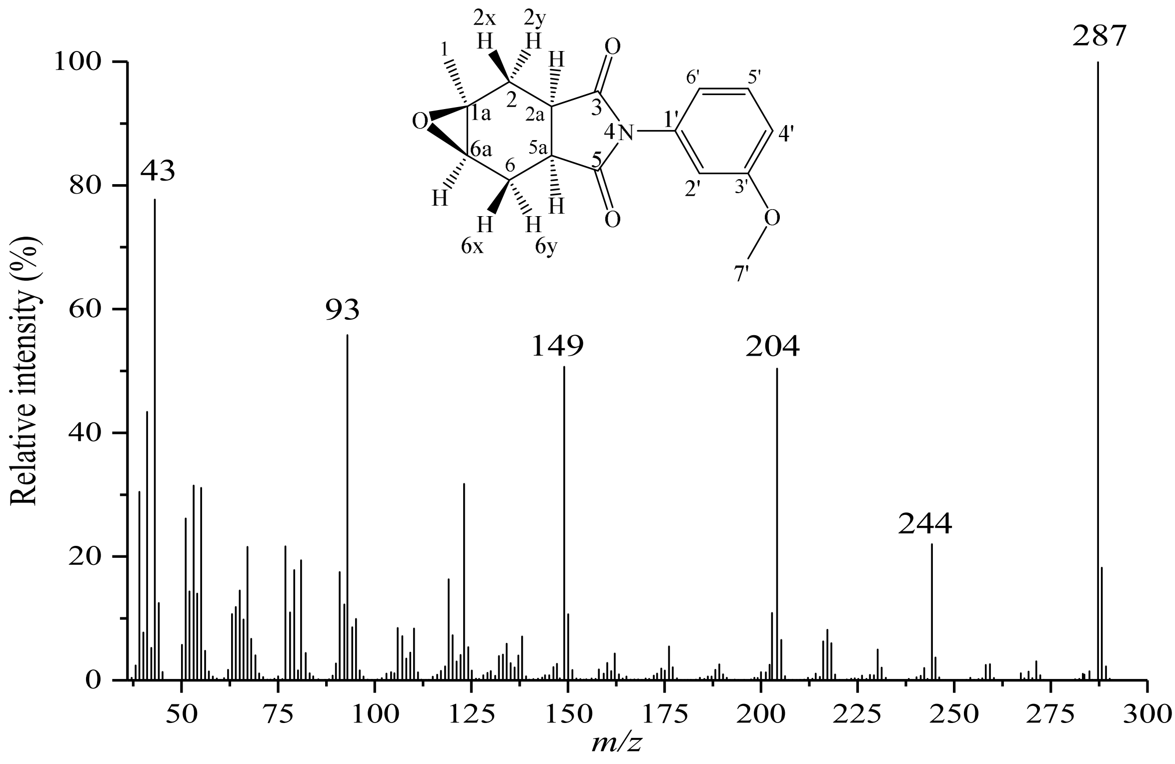

Figure S3 – Mass spectrum of compound **5b**.

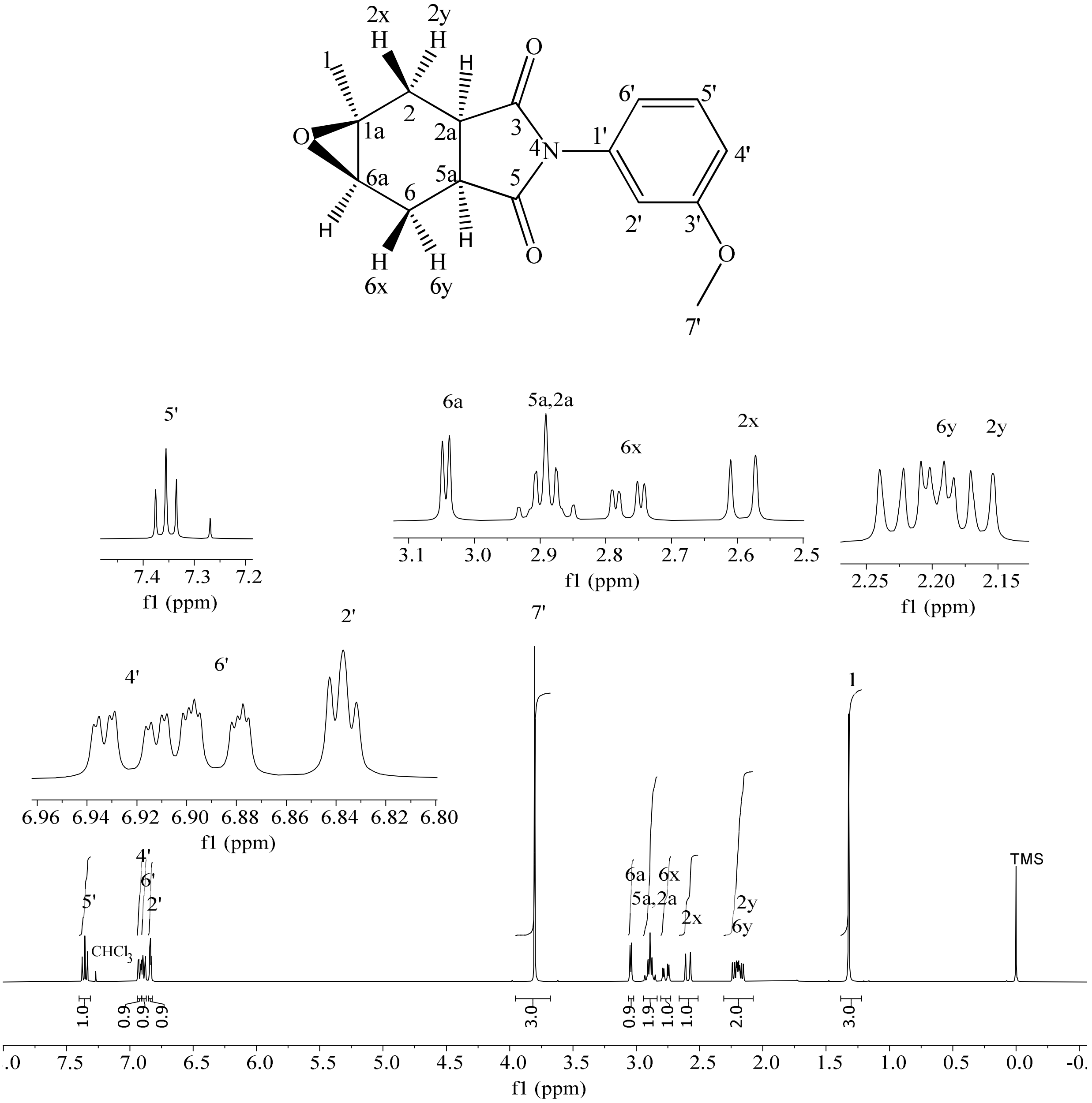

Figure S4 – ^{1}H NMR spectrum (400 MHz, $CDCl_3$ δ_{CHCl_3} = 7.27 ppm) of compound **5b**.

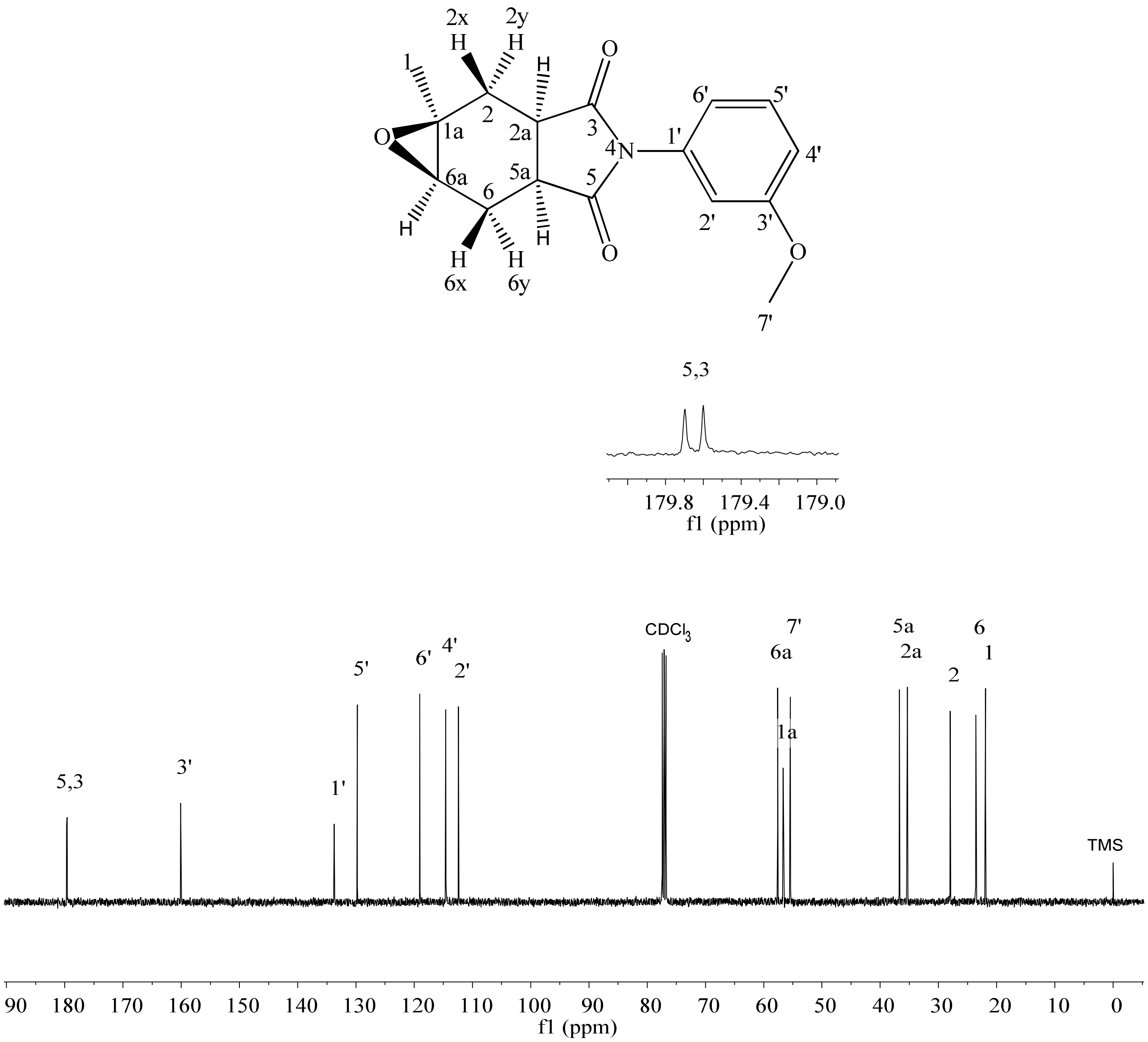

Figure S5 – ^{13}C NMR spectrum (100 MHz, $CDCl_3$ δ_{CDCl3} = 77.0 ppm) of compound **5b**.

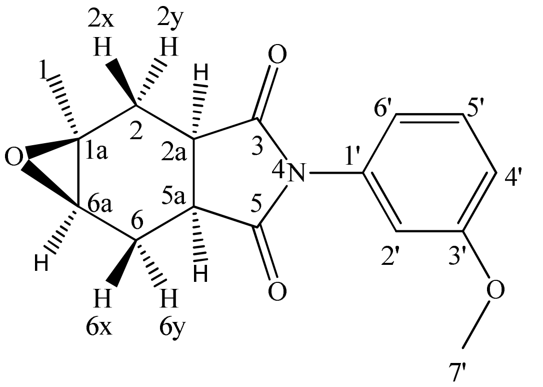

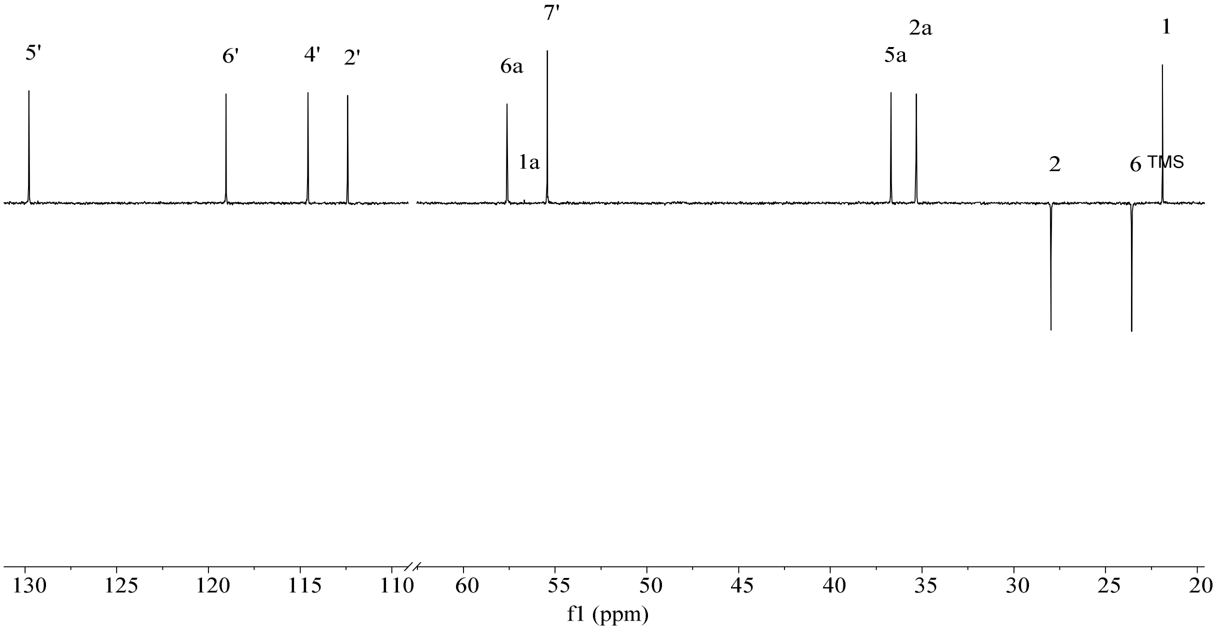

Figure S6– DEPT spectrum (100 MHz, $CDCl_3$ δ_{CDCl3} = 77.0 ppm) of compound **5b**.

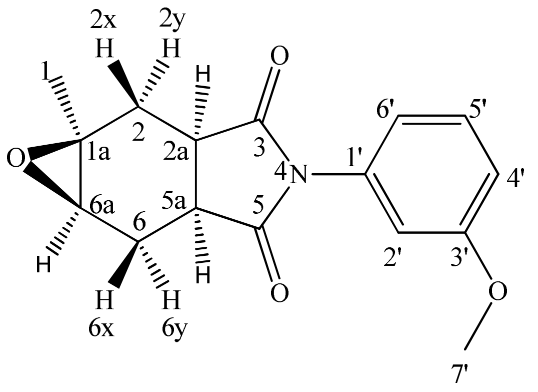

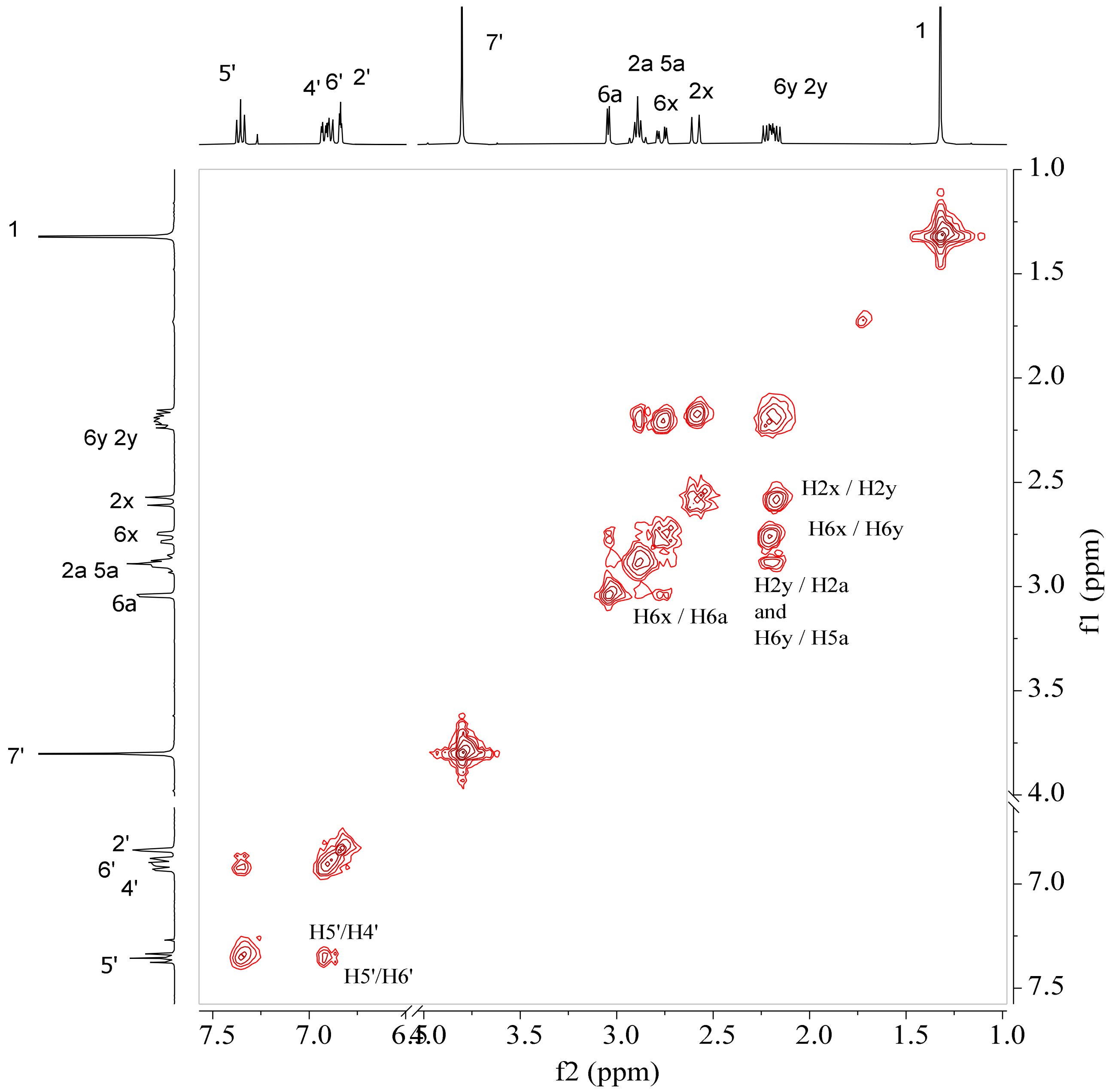

Figure S7 – COSY contour map of compound **5b**.

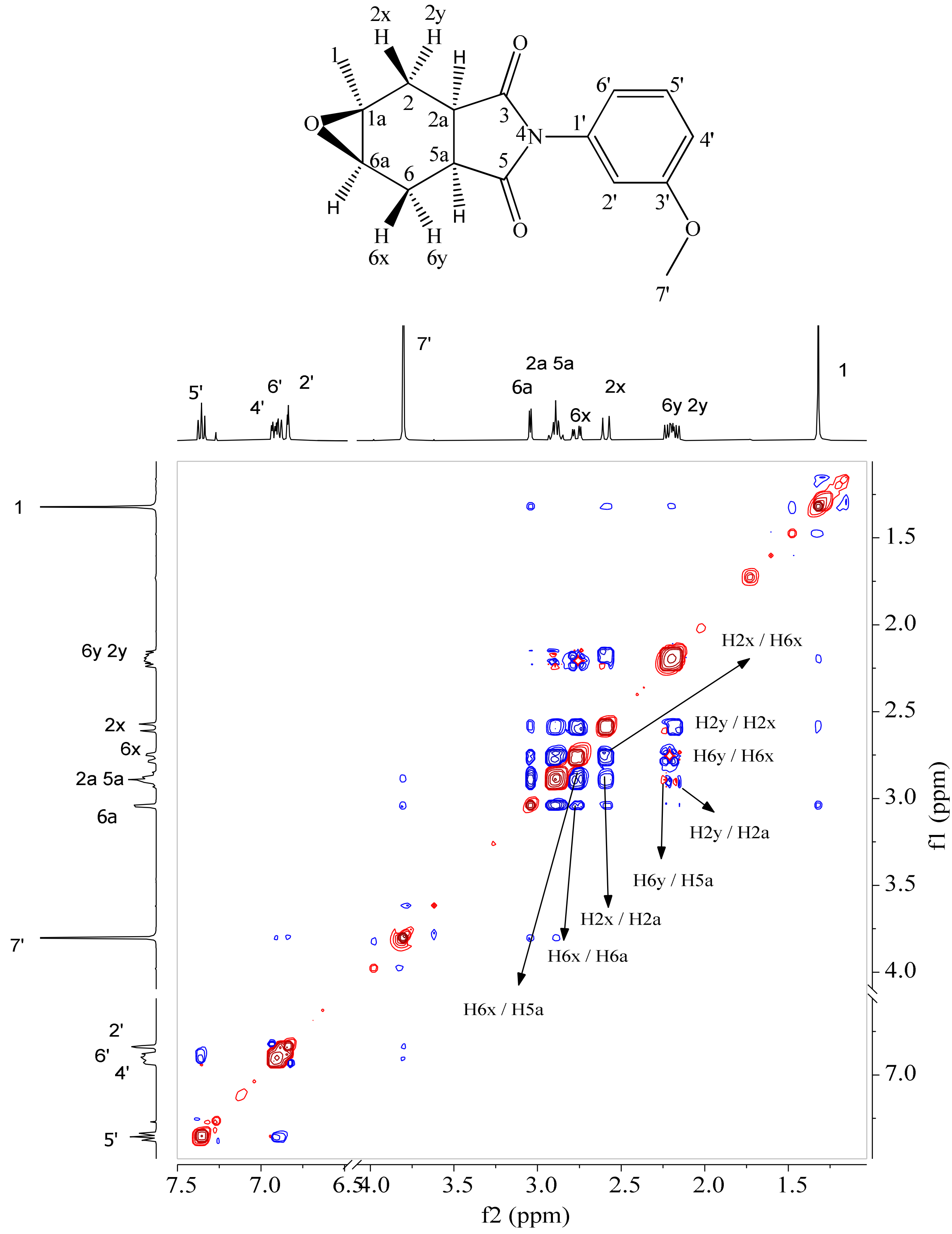

Figure S8 – NOESY contour map of compound **5b**.

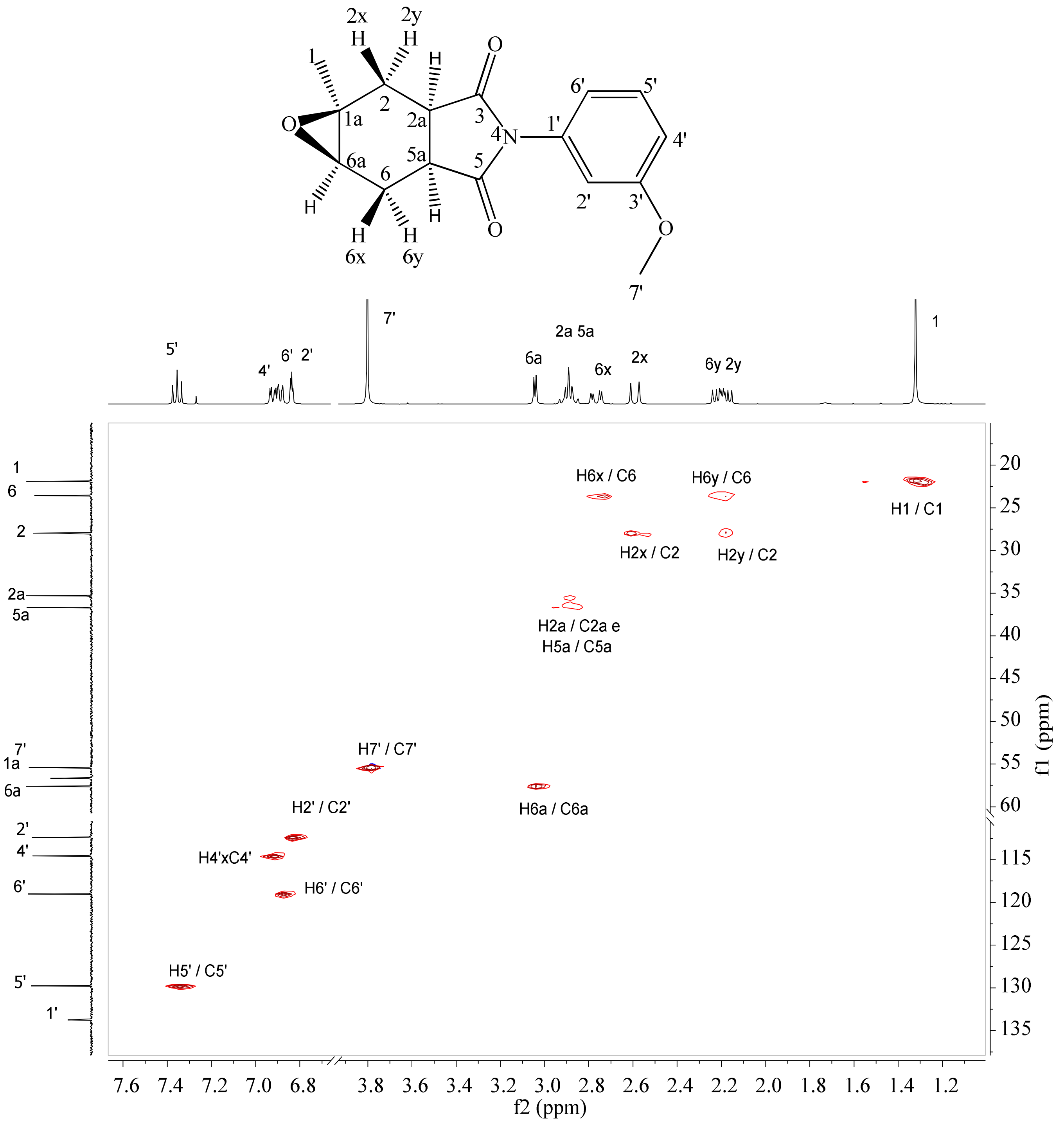

Figure S9 – HMQC contour map of compound **5b**.

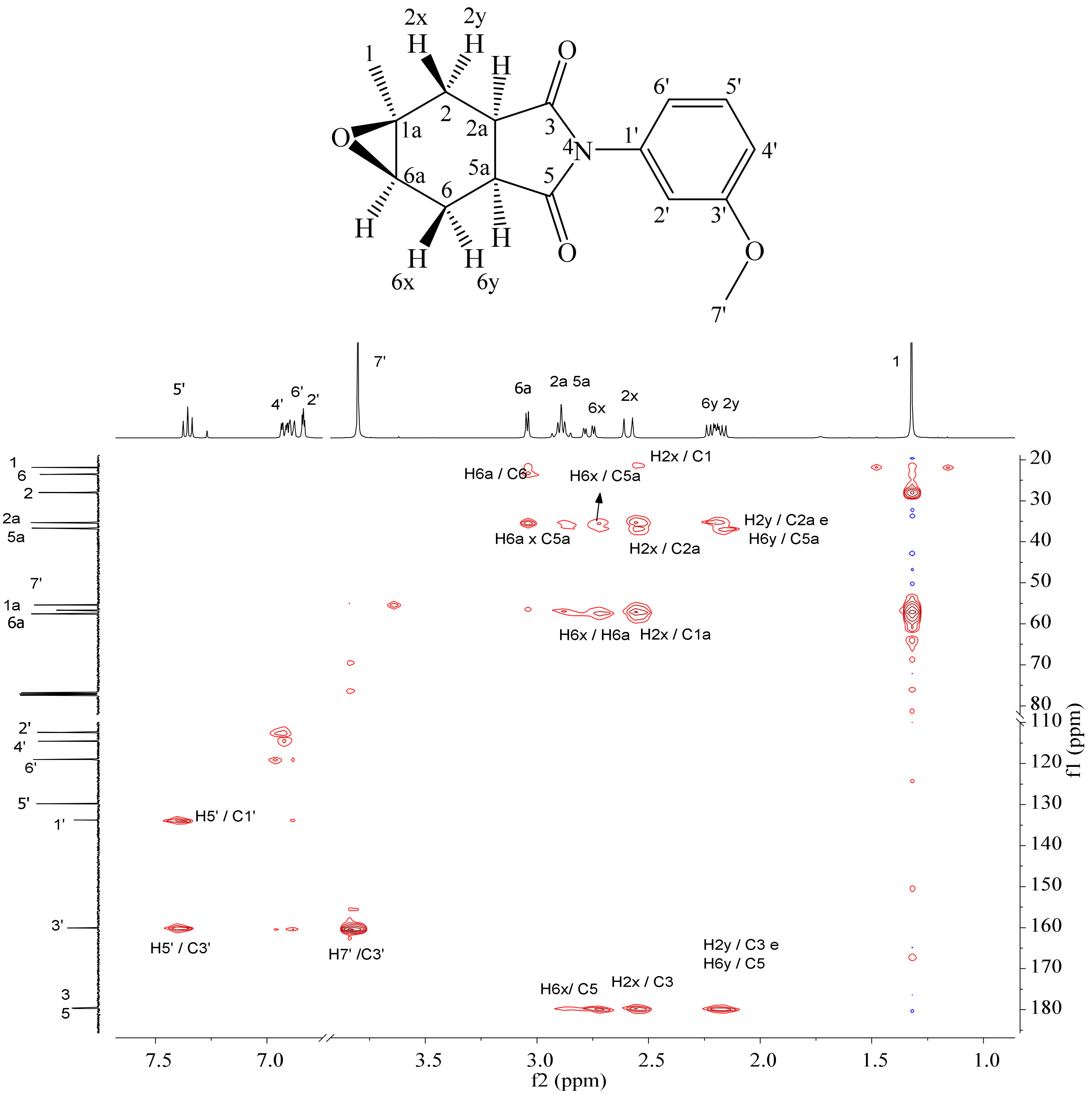

Figure S10 - HMBC contour map of compound **5b**.

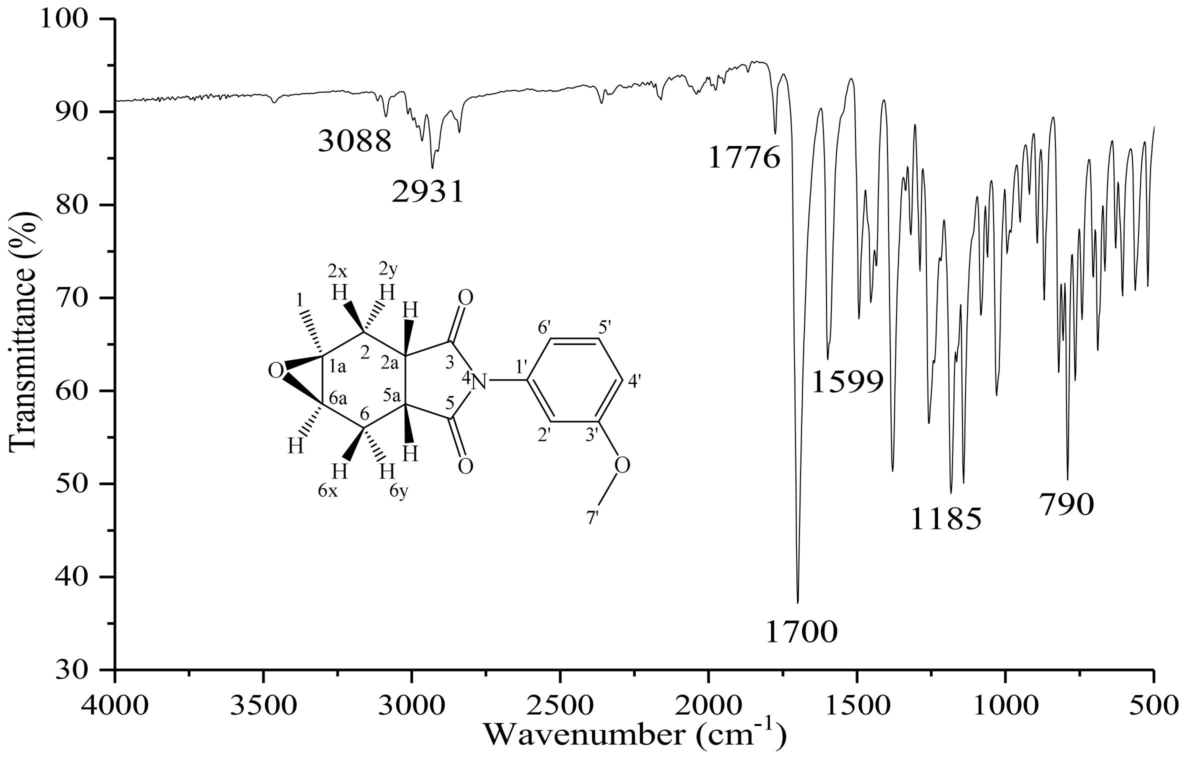

Figure S11 - IR spectrum of the compound **5a.**

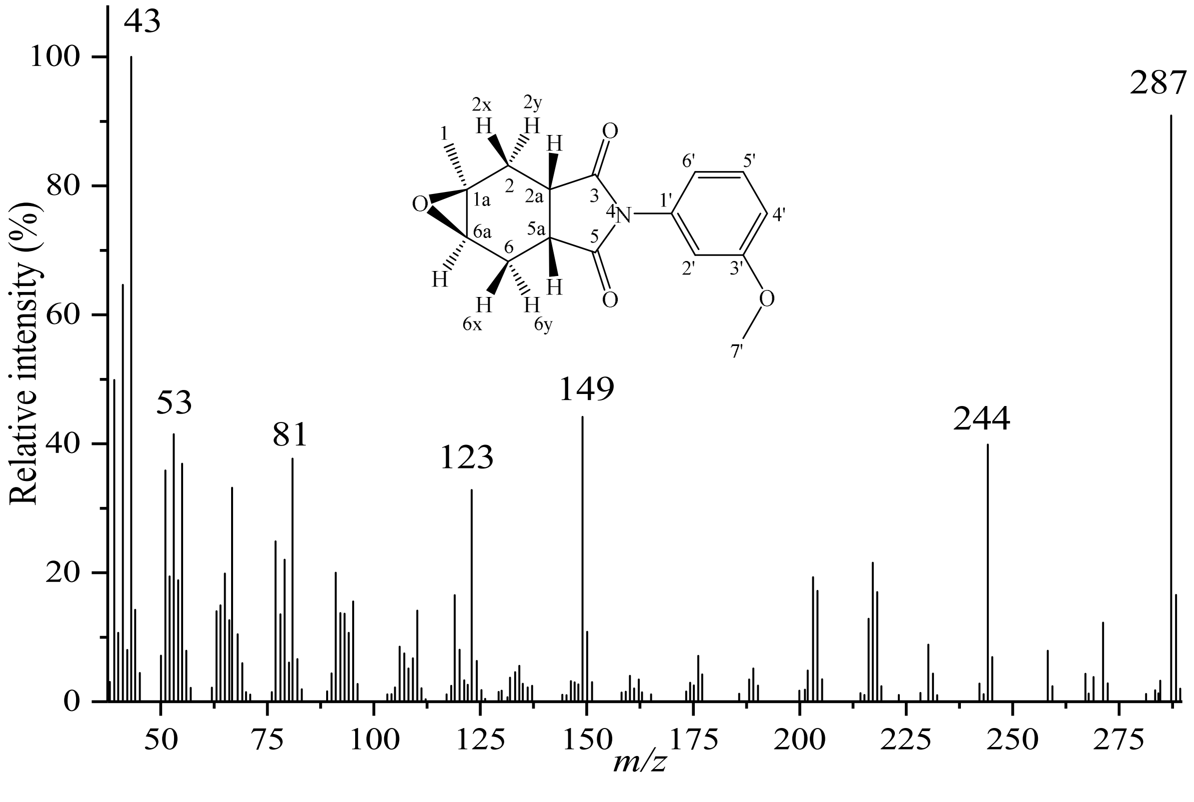

Figure S12- Mass spectrum of compound **5a**.

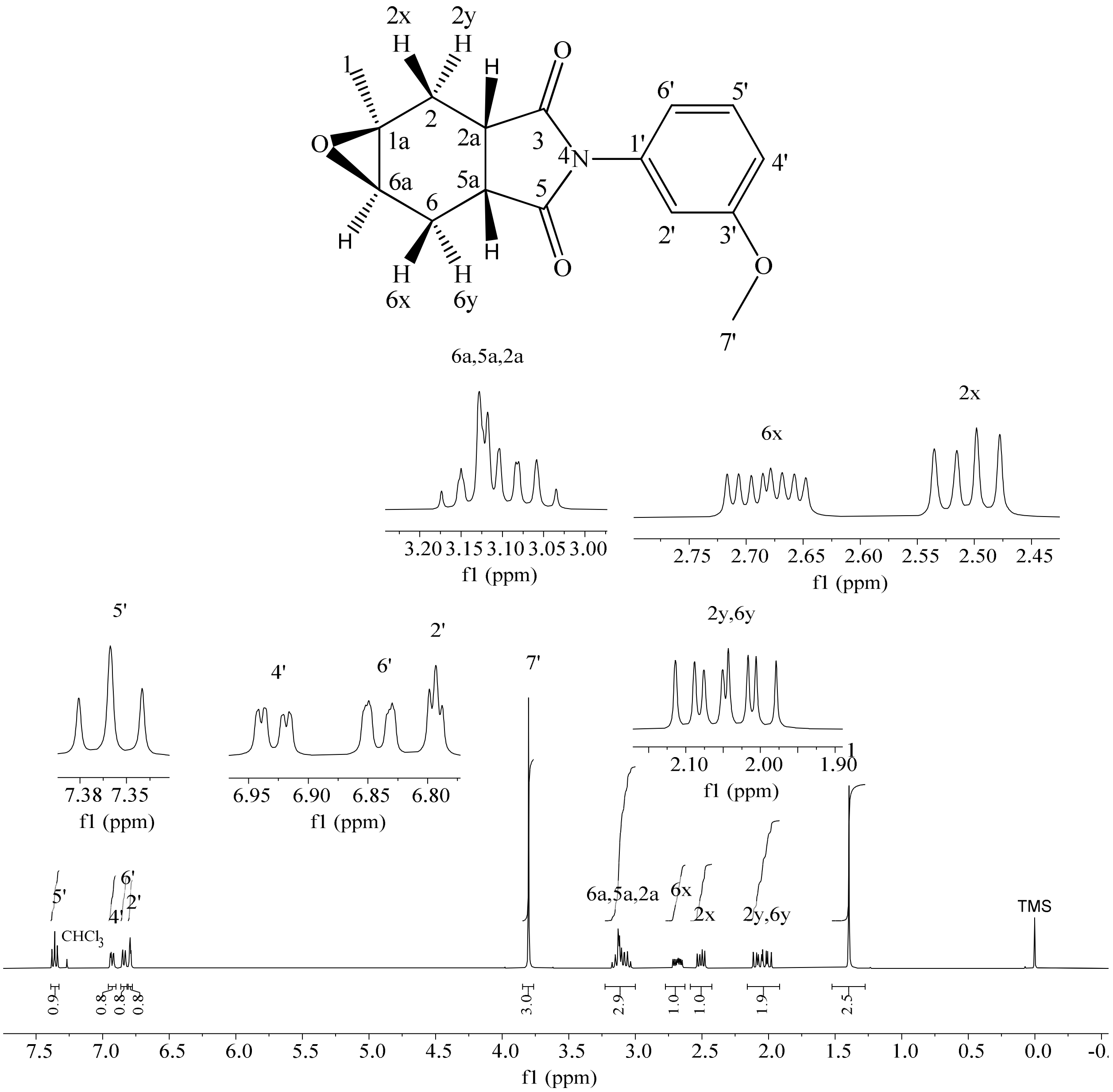

Figure S13- ^{1}H NMR spectrum (400 MHz, $CDCl_3$ δ_{CDCl3} = 7.27 ppm) of compound **5a**.

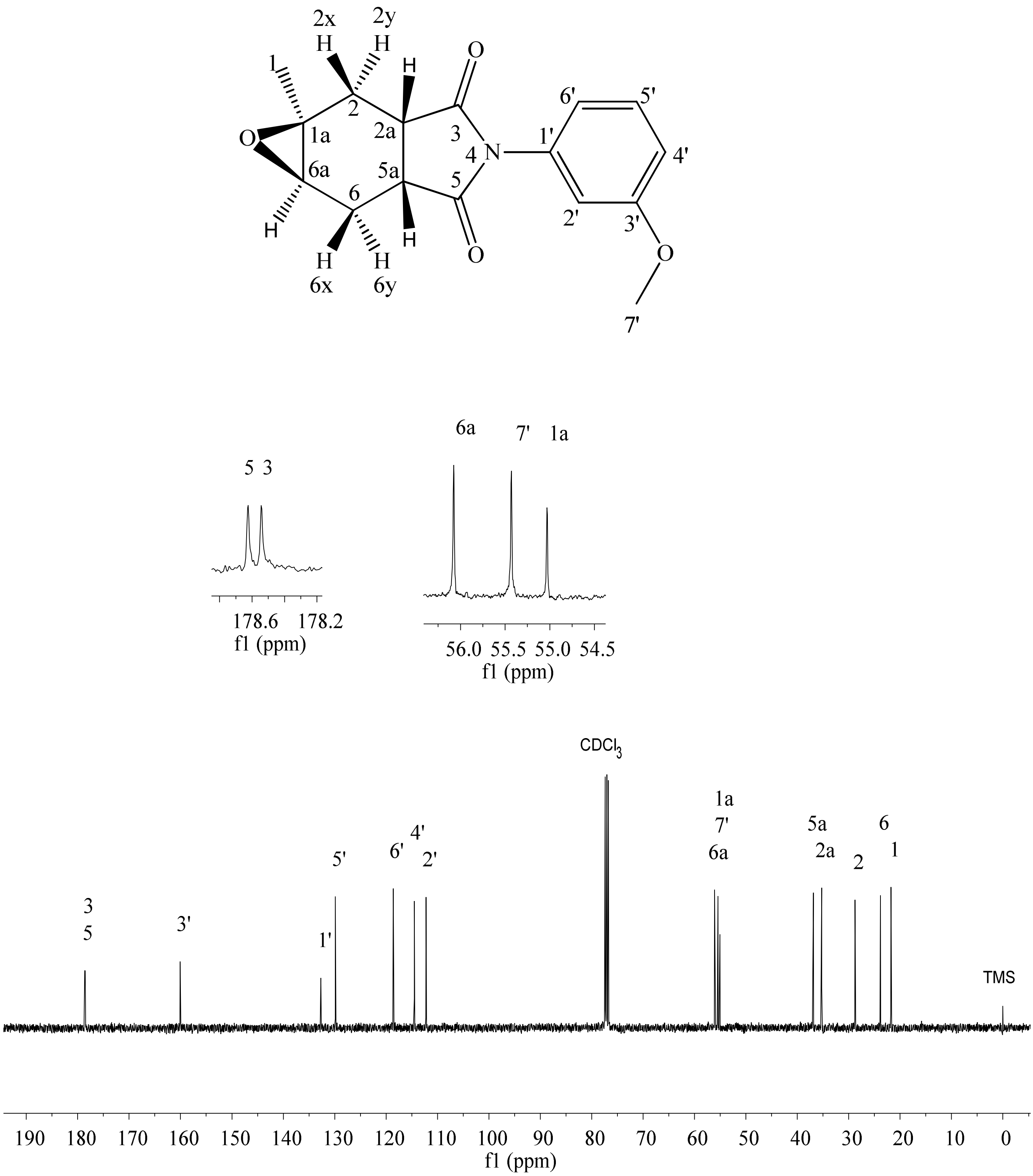

Figure S14 – ^{13}C NMR spectrum (101 MHz, $CDCl_3$ δ_{CDCl3} = 77.0 ppm) of compound **5a**.

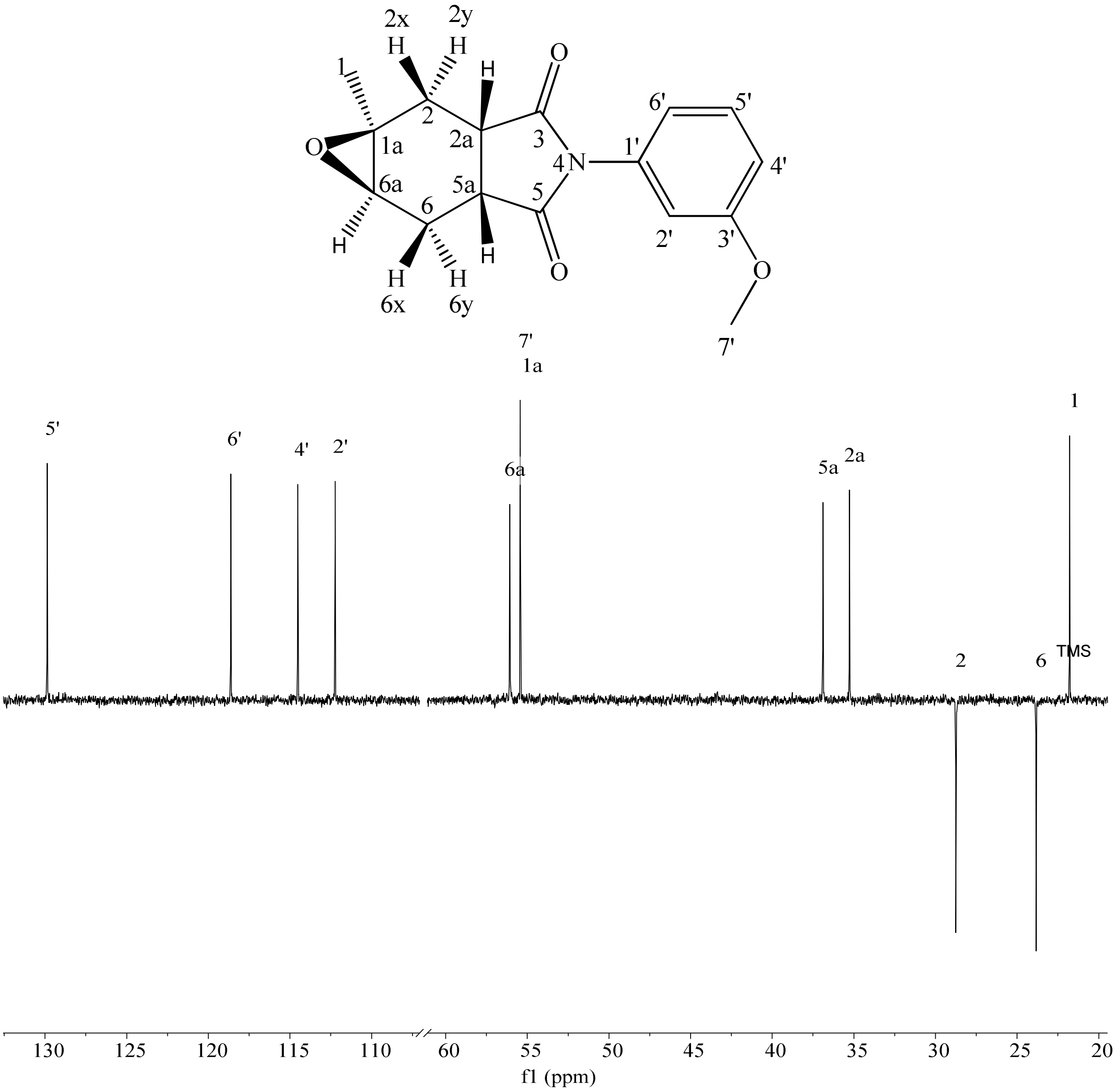

Figure S15 – DEPT spectrum (100 MHz, $CDCl_3$ δ_{CDCl3} = 77.0 ppm) of compound **5a**.

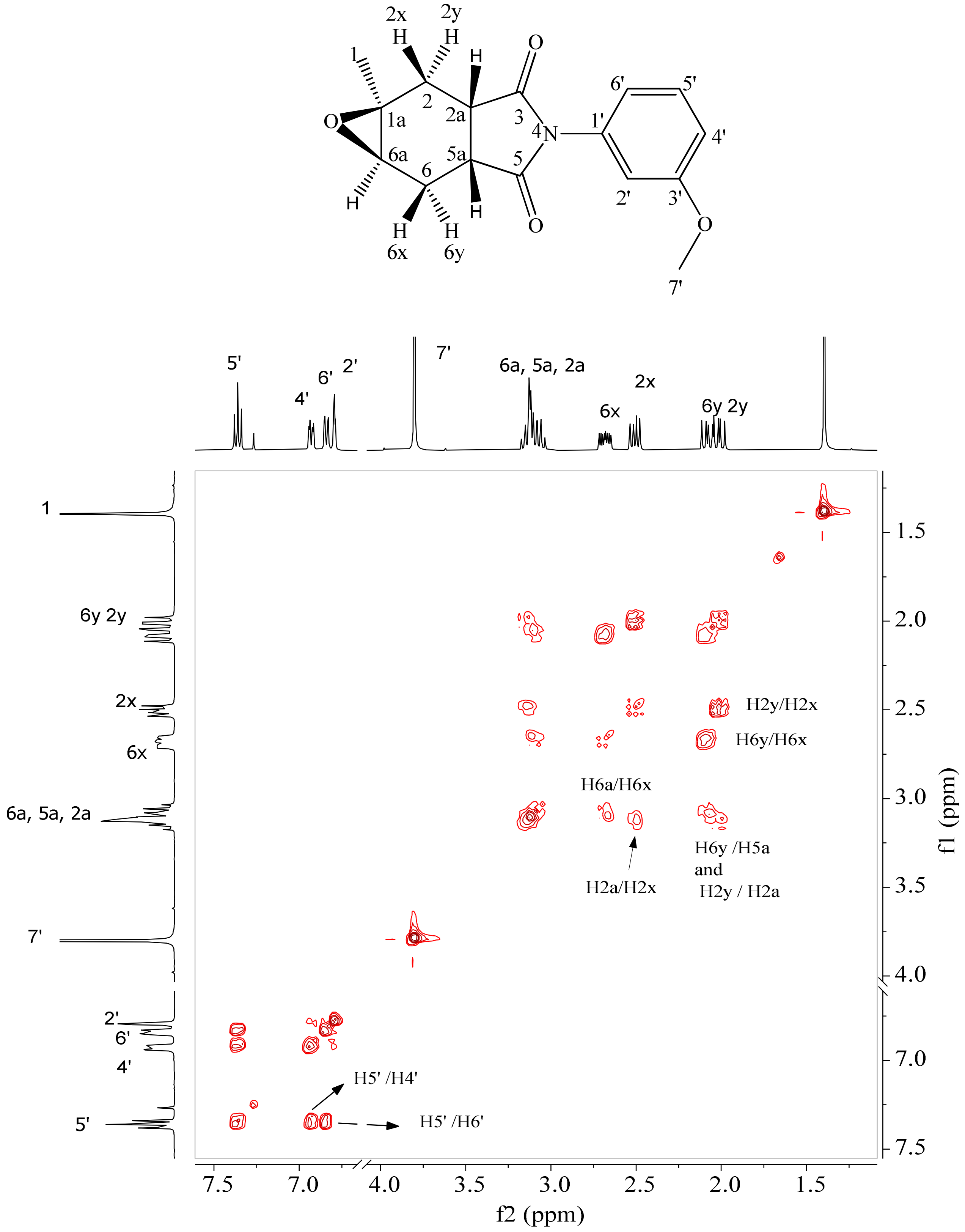

Figure S16 - COSY contour map of compound **5a**.

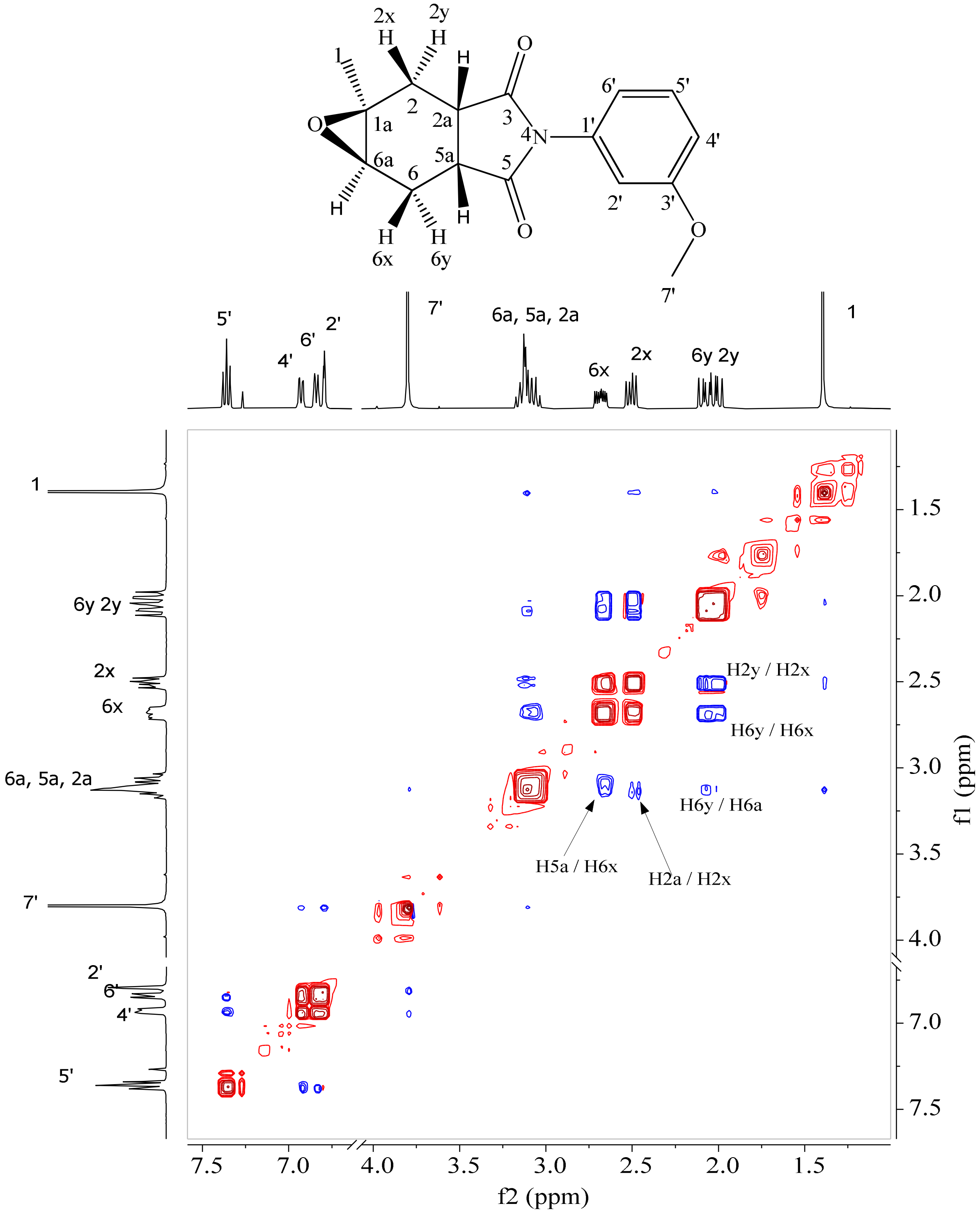

Figure S17 – NOESY contour map of compound **5a**.

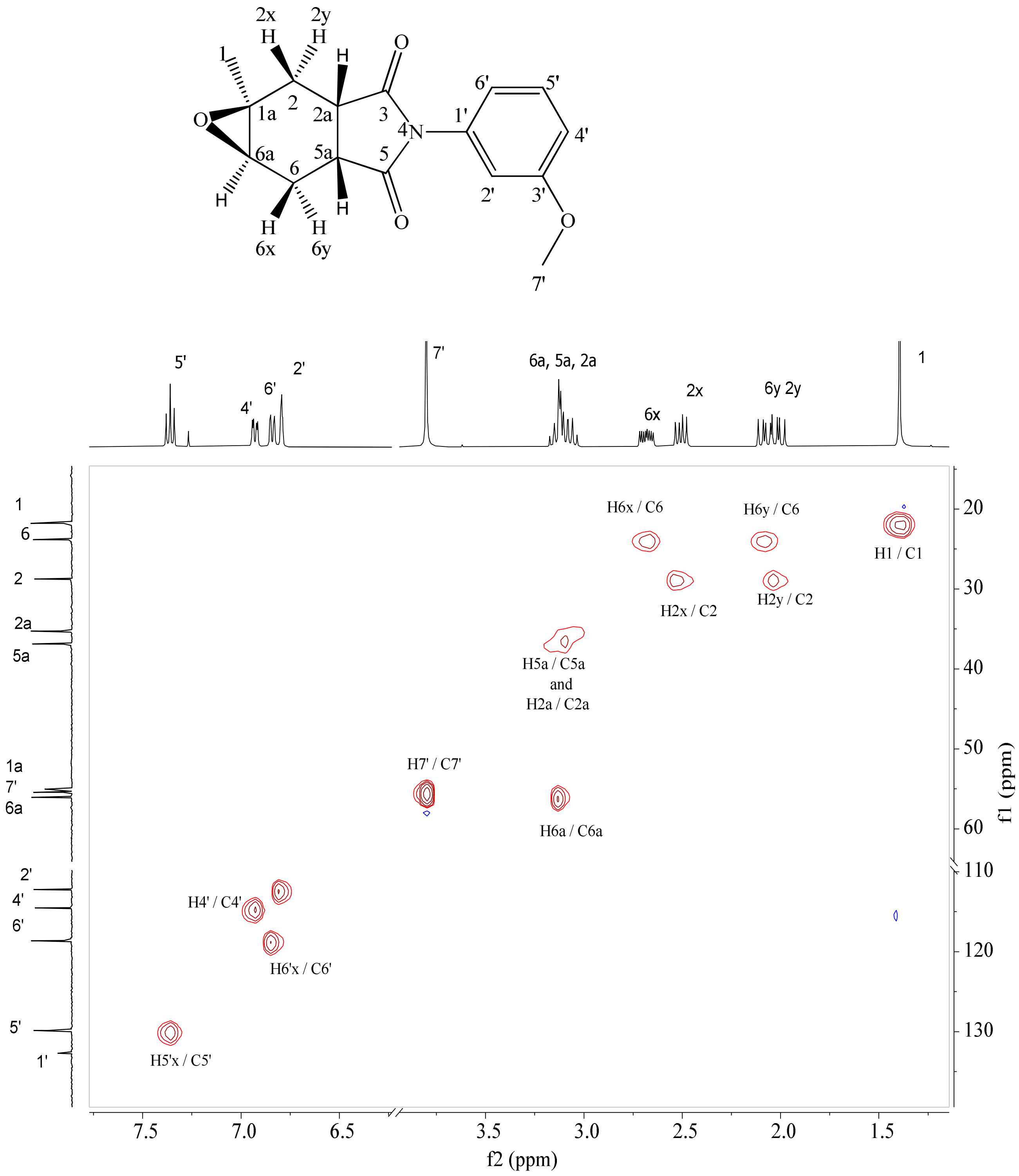

Figure S18– HMQC contour map of compound **5a**.

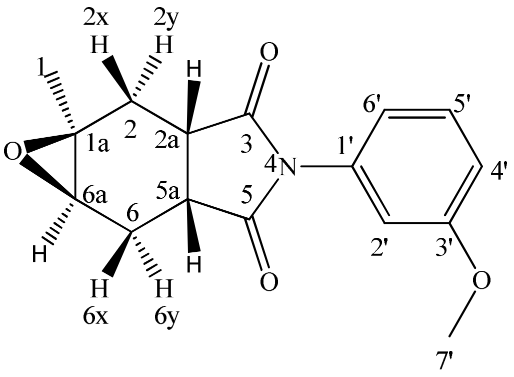

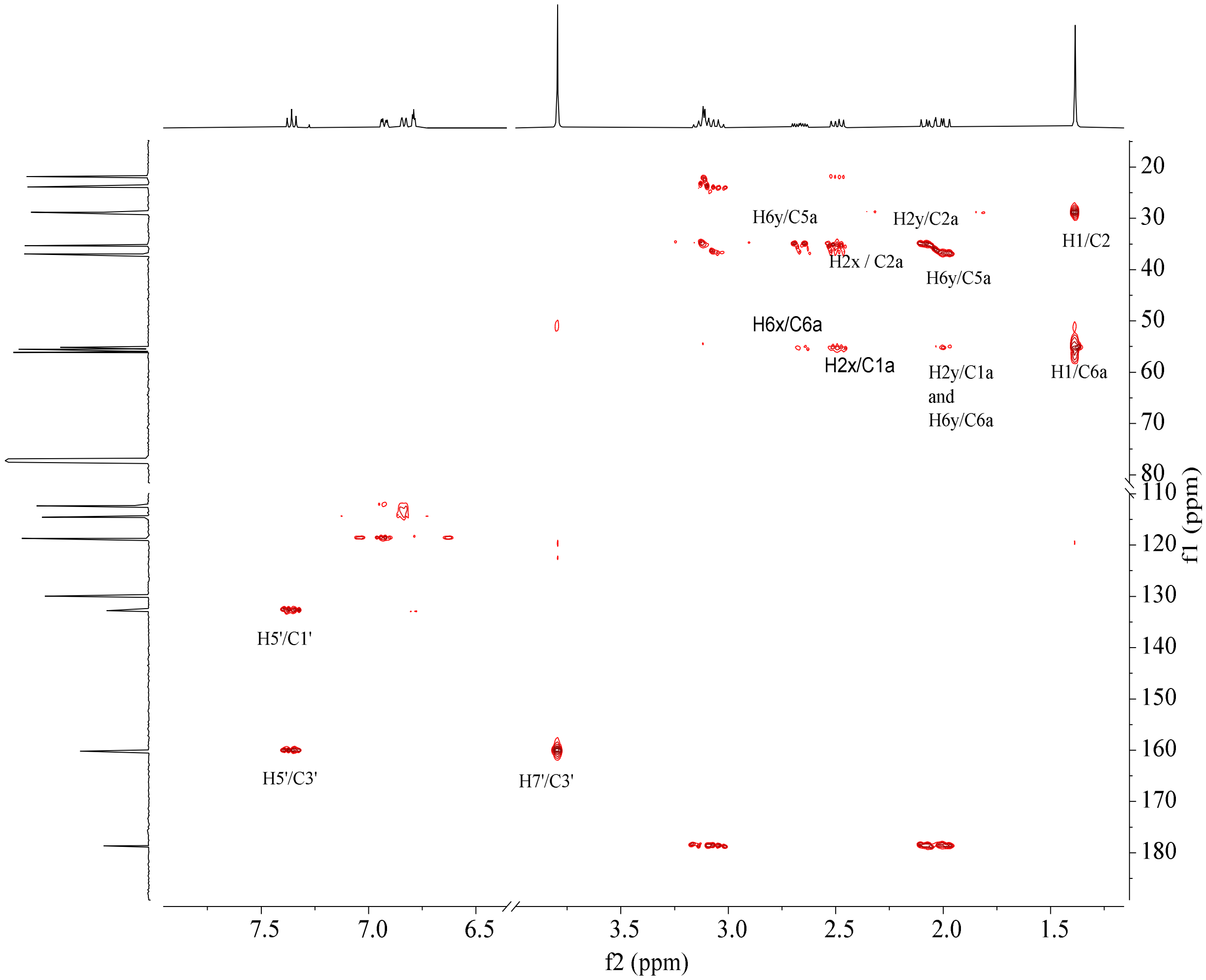

Figure S19 – HMBC contour map of compound **5a**.

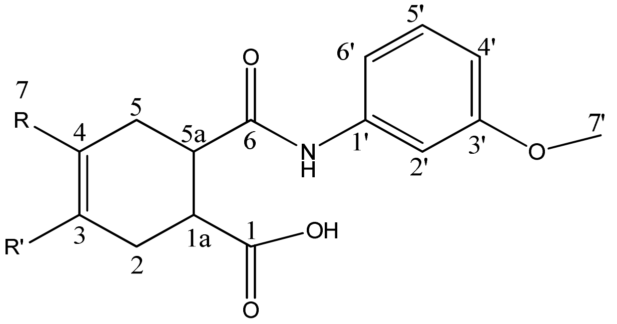

R = CH_3, R' = H
+
R = H, R' = CH_3

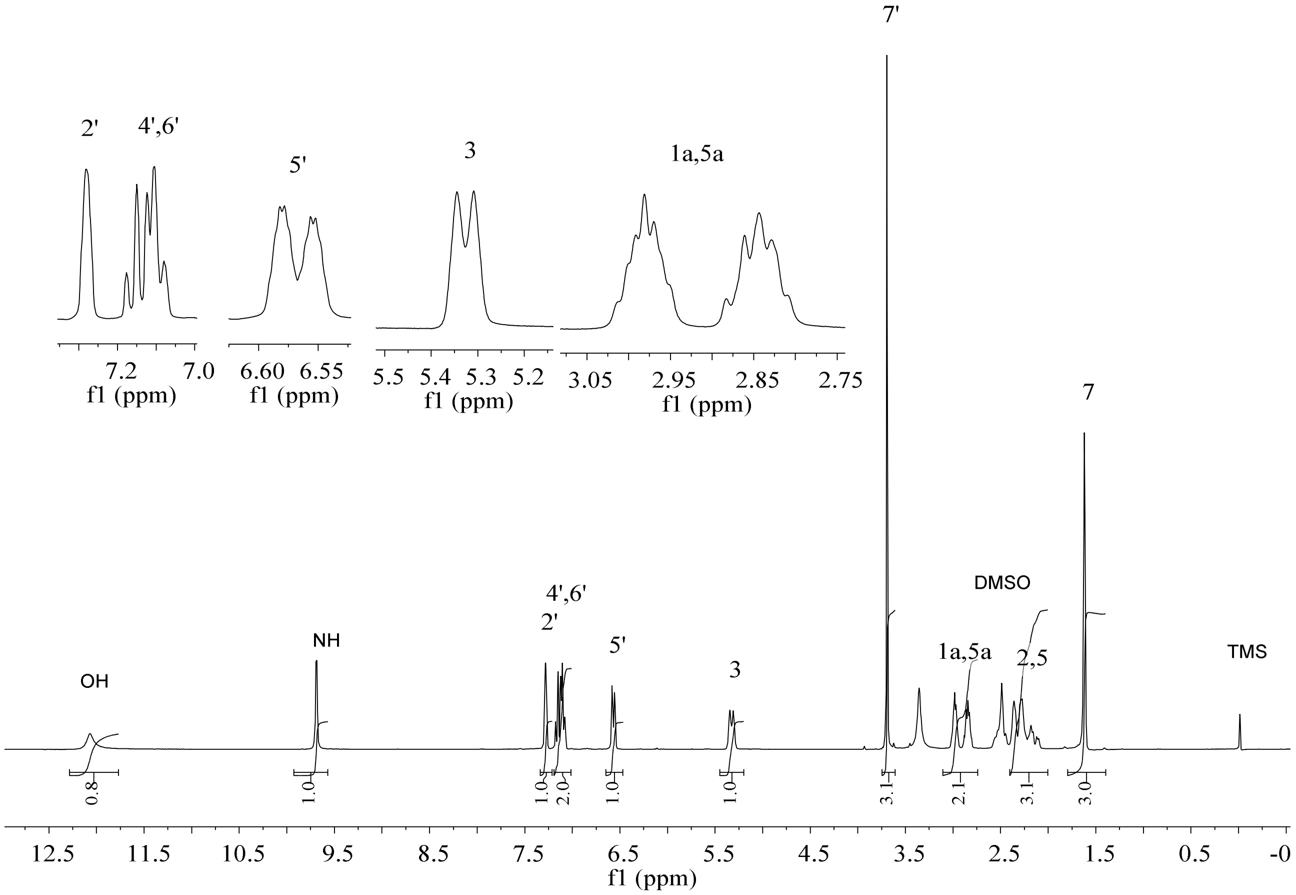

Figure S20 – ^{1}H NMR spectrum (300 MHz, DMSO, δ_{DMSO} = 2.50 ppm) of compound **2**.

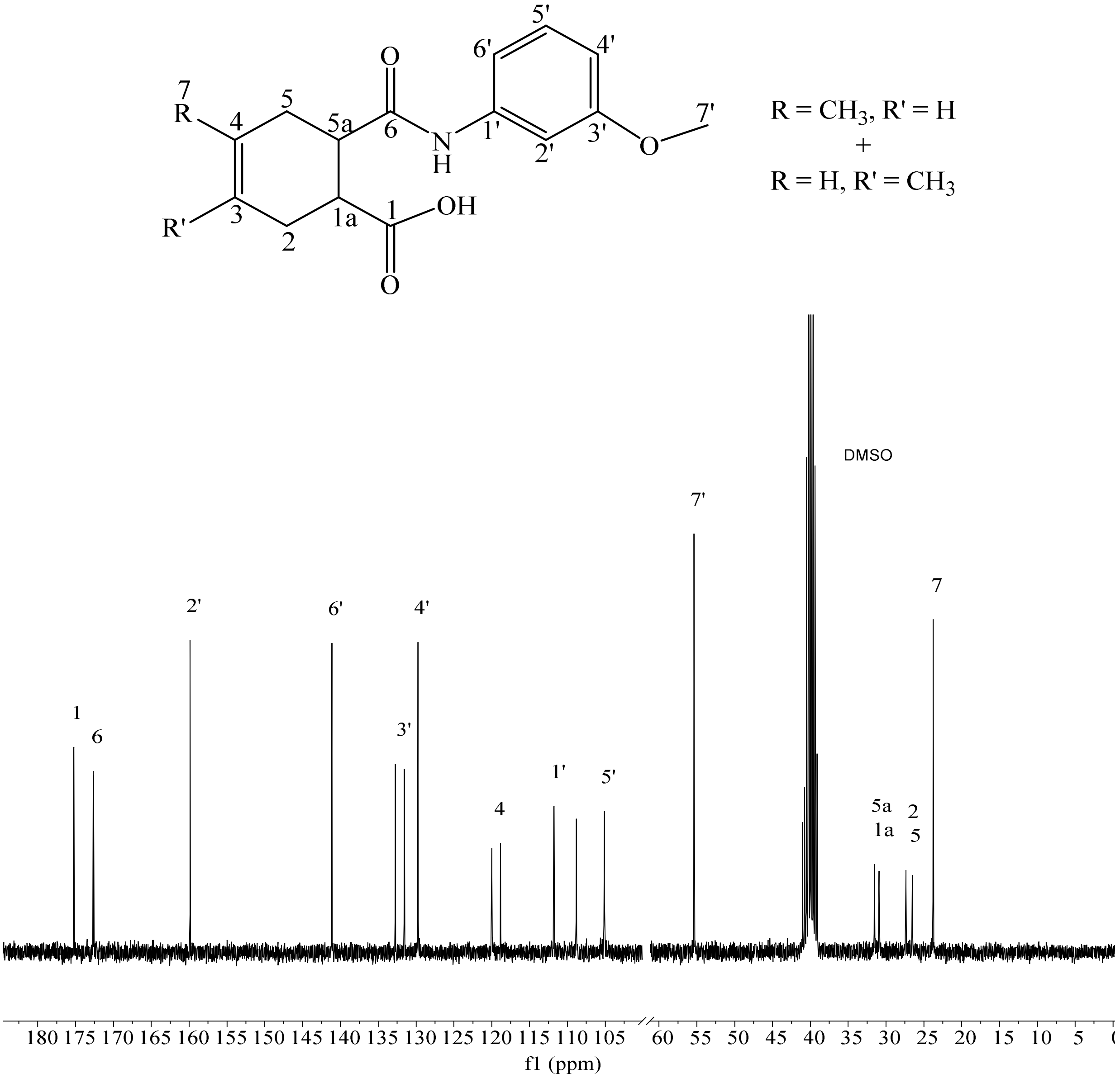

Figure S21 – ^{13}C NMR spectrum (75 MHz, DMSO, δ_{DMSO} = 40.0 ppm) of compound **2**.

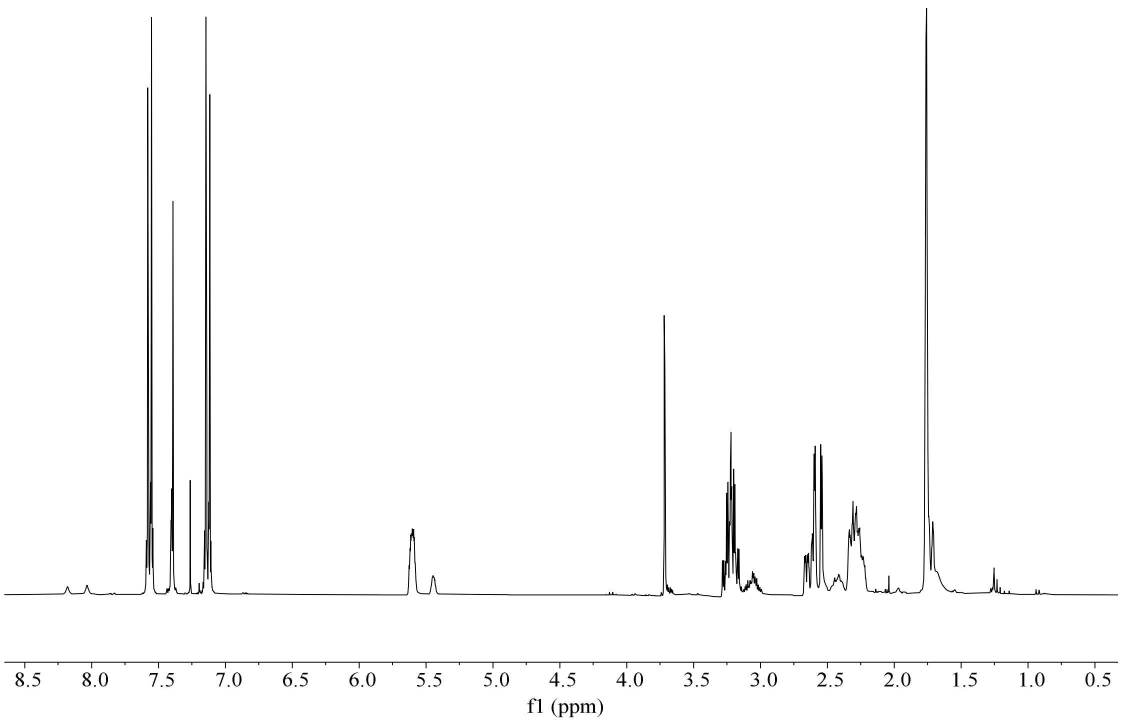

Figure S22 – ^{1}H NMR spectrum (300 MHz, $CDCl_3$ δ_{CDCl3} = 7.27 ppm) of the mixture of compounds **3** and **4**.

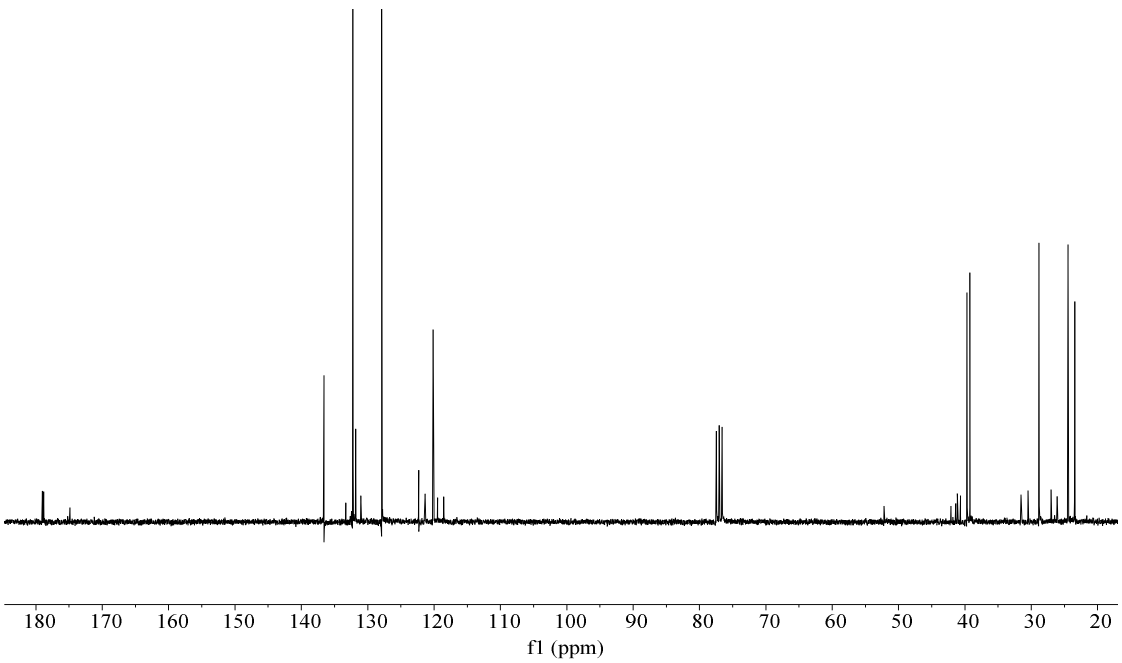

Figure S23 – ^{13}C NMR spectrum (75 MHz, $CDCl_3$ δ_{CDCl3} = 77.0 ppm) of the mixture of compounds **3** and **4**.

$$P(A_1 | B) = \frac{P(A_1) \times P(B | A_1)}{P(A_1)\, P(B | A_1) + P(A_2)\, P(B | A_2)}$$

Equation S1: Bayes´ theorem for calculating conditional probability. $P(A_1|B)$ is the probability, without previous NMR interpretation, that the proposed assignment combination (**5a**$_{\mathbf{exp}}$=**5a**$_{\mathbf{calc}}$, **5b**$_{\mathbf{exp}}$=**5b**$_{\mathbf{calc}}$) is correct.

Table S1: Development of lettuce seeds in relation to the control.

	Seeds development (% of control)					
	Substance					
**Concentration**	**5a-stalk**	**5b-stalk**	**Dual-stalk**	**5a-root**	**5b-root**	**Dual-root**
**500 µM**	-43	-17	-65	-52	-25	-74
**300 µM**	-40	-17	-56	-40	-50	-63
**150 µM**	-26	0	-53	-65	-20	-51
**100 µM**	-23	7	-35	-69	4	-22
**50 µM**	-15	-24	-37	-34	-49	-41

Table S2: Development of cucumber seeds in relation to the control.

	Seeds development (% of control)					
	Substance					
**Concentration**	**5a-stalk**	**5b-stalk**	**Dual-stalk**	**5a-root**	**5b-root**	**Dual-root**
**500 µM**	-20	-11	-50	-8	2	-57
**300 µM**	2	-7	-38	10	7	-51
**150 µM**	-13	-12	-29	8	3	-48
**100 µM**	-8	-2	-24	10	15	-50
**50 µM**	-8	-3	-19	10	1	-48

Table S3: Development of sorghum seeds in relation to control.

	Seeds development (% of control)					
	Substance					
**Concentration**	**5a-stalk**	**5b-stalk**	**Dual-stalk**	**5a-root**	**5b-root**	**Dual-root**
**500 µM**	-49	-38	-64	-48	-46	-79
**300 µM**	-45	-52	-56	-45	-47	-58
**150 µM**	-62	-32	-34	-55	-23	-45
**100 µM**	-51	-29	-52	-49	-37	-50
**50 µM**	-51	-27	-53	-43	-25	-63

Table S4: Development of beggartick seeds in relation to control.

	Seeds development (% of control)					
	Substance					
**Concentration**	**5a-stalk**	**5b-stalk**	**Dual-stalk**	**5a-root**	**5b-root**	**Dual-root**
**500 µM**	-61	11	-77	-86	-81	-91
**300 µM**	-16	-24	-67	-77	-85	-84
**150 µM**	-11	-1	-49	-76	-74	-73
**100 µM**	-28	-1	-56	-70	-80	-79
**50 µM**	-10	-41	-48	-72	-84	-76

Table S5: CMAE analyses calculated after linear correction of the calculated ^{13}C NMR chemical shifts.

**Product 5a+5b**					
**5a+5a** / **5b+5b**		δ_C Calc **5a+5b**	δ_C Expt **5a+5b**	δ_C Calc **5a+5b** corrected	$\|\Delta\delta\|$
1		23.15	21.79	18.99	2.80
6		29.82	23.83	25.52	1.69
2		34.41	28.75	30.03	1.28
2a		42.48	35.27	37.92	2.65
5a		43.82	36.89	39.24	2.35
7'		57.21	55.03	52.34	2.69
1A		59.02	55.43	54.12	1.31
6a		60.28	56.08	55.35	0.73
2'		117.97	112.23	111.85	0.38
4'		118.53	114.52	112.40	2.12
6'		124.17	118.62	117.92	0.70
5'		135.64	129.87	129.15	0.72
1'		141.10	132.71	134.49	1.78
3'		168.40	160.09	161.23	1.14
C=O		189.79	178.54	182.18	3.64
C=O		189.88	178.62	182.26	3.64
1		23.32	21.89	19.17	2.72
6		28.85	23.56	24.58	1.02
2		32.85	27.96	28.49	0.53
2a		42.71	35.31	38.15	2.84
5a		43.98	36.68	39.39	2.71
7'		57.16	55.42	52.30	3.12
1A		61.50	56.68	56.55	0.13
6a		62.56	57.61	57.59	0.02
2'		118.07	112.40	111.95	0.45
4'		118.50	114.57	112.36	2.21
6'		124.77	119.03	118.50	0.53
5'		135.67	129.78	129.18	0.60
1'		142.37	133.76	135.74	1.98
3'		168.52	160.09	161.35	1.26
C=O		190.75	179.60	183.12	3.52
C=O		190.78	179.70	183.15	3.45
r	0.999	Intercept	-3.139		
r^2	0.999	Slope	0.971	CMAE[a]	1.66

[a]The linear correction was done using the ^{13}C NMR chemical shifts of **5a+5b**. The CMAE was calculated using (**5a+5b**)calc vs. (**5a+5b**)exp.

Table S6: CMAE analyses calculated after linear correction of the calculated ^{13}C NMR chemical shifts.

Product 5a+5b					
**5a+5b** / **5b+5a**		δ_C Calc **5b+5a**	δ_C Expt **5a+5b**	δ_C Calc **5b+5a** corrected	$\lvert\Delta\delta\rvert$
1		23.32	21.79	19.18	2.61
6		28.85	23.83	24.59	0.76
2		32.85	28.75	28.50	0.25
2a		42.71	35.27	38.16	2.89
5a		43.98	36.89	39.40	2.51
7'		57.16	55.03	52.30	2.73
1A		61.50	55.43	56.55	1.12
6a		62.56	56.08	57.59	1.51
2'		118.07	112.23	111.94	0.29
4'		118.50	114.52	112.36	2.16
6'		124.77	118.62	118.50	0.12
5'		135.67	129.87	129.17	0.70
1'		142.37	132.71	135.73	3.02
3'		168.52	160.09	161.34	1.25
C=O		190.75	178.54	183.10	4.56
C=O		190.78	178.62	183.13	4.51
1		23.15	21.89	19.00	2.89
6		29.82	23.56	25.53	1.97
2		34.41	27.96	30.03	2.07
2a		42.48	35.31	37.93	2.62
5a		43.82	36.68	39.25	2.57
7'		57.21	55.42	52.35	3.07
1A		59.02	56.68	54.13	2.55
6a		60.28	57.61	55.36	2.25
2'		117.97	112.40	111.84	0.56
4'		118.53	114.57	112.39	2.18
6'		124.17	119.03	117.91	1.12
5'		135.64	129.78	129.14	0.64
1'		141.10	133.76	134.49	0.73
3'		168.40	160.09	161.22	1.13
C=O		189.79	179.60	182.16	2.56
C=O		189.88	179.70	182.25	2.55
r	0.999	Intercept	-2.991		
r²	0.998	Slope	0.968	CMAE[a]	1.91

[a]The linear correction was done using the ^{13}C NMR chemical shifts of **5a+5b**. The CMAE was calculated using (**5b+5a**)calc vs. (**5a+5b**)exp.

Table S7: CMAE analyses calculated after linear correction of the calculated ^{1}H NMR chemical shifts.

Product 5a+5b					
**5a+5a** / **5b+5b**		δ_H Calc **5a+5b**	δ_H Expt **5a+5b**	δ_H Calc **5a+5b** corrected	$\lvert\Delta\delta\rvert$
1		1.26	1.40	1.40	0.00
2y		2.02	2.01	2.11	0.10
6y		2.13	2.08	2.21	0.13
2x		2.22	2.50	2.28	0.22
6x		2.52	2.68	2.57	0.11
2a		3.00	3.06	3.01	0.05
5a		3.10	3.13	3.10	0.03
6a		3.12	3.14	3.12	0.02
7’		3.88	3.80	3.82	0.02
2’		7.14	6.79	6.84	0.05
6’		7.15	6.84	6.84	0.00
4’		7.25	6.93	6.93	0.00
5’		7.70	7.36	7.35	0.01
1		1.20	1.32	1.35	0.03
2y		2.17	2.18	2.25	0.07
6y		2.19	2.22	2.26	0.04
2x		2.58	2.59	2.62	0.03
6x		2.79	2.77	2.81	0.04
2a		2.85	2.89	2.87	0.02
5a		2.89	2.89	2.91	0.02
6a		2.95	3.04	2.96	0.08
7’		3.88	3.80	3.82	0.02
2’		7.11	6.84	6.81	0.03
6’		7.21	6.89	6.90	0.01
4’		7.24	6.92	6.93	0.01
5’		7.70	7.36	7.35	0.01
r	1.000	Intercept	0.237		
r^2	0.999	Slope	0.924	CMAE[a]	0.04

[a]The linear correction was done using the ^{1}H NMR chemical shifts of **5a+5b**. The CMAE was calculated using (**5a+5b**)calc vs. (**5a+5b**)exp.

Table S8: CMAE analyses calculated after linear correction of the calculated ^{1}H NMR chemical shifts.

**Product 5a+5b**				
**5a+5b** / **5b+5a**	δ_H Calc **5b+5a**	δ_H Expt **5a+5b**	δ_H Calc **5b+5a** corrected	$\lvert\Delta\delta\rvert$
1	1.20	1.40	1.35	0.05
6	2.17	2.01	2.25	0.24
2	2.19	2.08	2.26	0.18
2a	2.58	2.50	2.63	0.13
5a	2.79	2.68	2.81	0.13
7’	2.85	3.06	2.87	0.19
1A	2.89	3.13	2.91	0.22
6a	2.95	3.14	2.96	0.18
2’	3.88	3.80	3.83	0.03
4’	7.11	6.79	6.80	0.01
6’	7.21	6.84	6.90	0.06
5’	7.24	6.93	6.92	0.01
1’	7.70	7.36	7.35	0.01
1	1.26	1.32	1.41	0.09
6	2.02	2.18	2.11	0.07
2	2.13	2.22	2.21	0.01
2a	2.22	2.59	2.29	0.30
5a	2.52	2.77	2.57	0.20
7’	3.00	2.89	3.01	0.12
1A	3.10	2.89	3.10	0.21
6a	3.12	3.04	3.12	0.08
2’	3.88	3.80	3.82	0.02
4’	7.14	6.84	6.83	0.01
6’	7.15	6.89	6.84	0.05
5’	7.25	6.92	6.93	0.01
1’	7.70	7.36	7.35	0.01
r 0.998	Intercept	0.242		
r^2 0.996	Slope	0.923	CMAE[a]	0.10

[a]The linear correction was done using the ^{1}H NMR chemical shifts of **5a+5b**. The CMAE was calculated using (**5b+5a**)calc vs. (**5a+5b**)exp.

1, 6, 2, 2a, 5a, 7’, 1A, 6a, 2’, 4’, 6’, 5’, 1’, 3’, C=O, C=O

Expt 5a

21.79, 23.83, 28.75, 35.27, 36.89, 55.03, 55.43, 56.08, 112.23, 114.52, 118.62, 129.87, 132.71, 160.09, 178.54, 178.62

Expt 5b

21.89, 23.56, 27.96, 35.31, 36.68, 55.42, 56.68, 57.61, 112.40, 114.57, 119.03, 129.78, 133.76, 160.09, 179.60, 179.70

Calc 5a

23.15, 29.82, 34.41, 42.48, 43.82, 57.21, 59.02, 60.28, 117.97, 118.53, 124.17, 135.64, 141.10, 168.40, 189.79, 189.88

Calc. 5b

23.32, 28.85, 32.85, 42.71, 43.98, 57.16, 61.50, 62.56, 118.07, 118.50, 124.77, 135.67, 142.37, 168.52, 190.75, 190.78

1, 2y, 6y, 2x, 6x, 2a, 5a, 6a, 7’, 2’, 6’, 4’, 5’

Expt 5a

1.40, 2.01, 2.08, 2.50, 2.68, 3.06, 3.13, 3.14, 3.80, 6.79, 6.84, 6.93, 7.36

Expt 5b

1.32, 2.18, 2.22, 2.59, 2.77, 2.89, 2.89, 3.04, 3.80, 6.84, 6.89, 6.92, 7.36

Calc. 5a

1.26, 2.02, 2.13, 2.22, 2.52, 3.00, 3.10, 3.12, 3.88, 7.14, 7.15, 7.25, 7.70

Calc. 5b

1.20, 2.17, 2.19, 2.58, 2.79, 2.85, 2.89, 2.95, 3.88, 7.11, 7.21, 7.24, 7.70

Enter two 1H and 13C experimental spectra then two calculated spectra:

13C Expt A	21.79, 23.83, 28.75, 35.27, 36.89, 55.03, 55.43, 56.08, 112.23, 114.52, 118.62, 129.87, 132.71, 160.09, 178.54, 178.62
13C Expt B	21.89, 23.56, 27.96, 35.31, 36.68, 55.42, 56.68, 57.61, 112.40, 114.57, 119.03, 129.78, 133.76, 160.09, 179.60, 179.70
13C Calc A	23.15, 29.82, 34.41, 42.48, 43.82, 57.21, 59.02, 60.28, 117.97, 118.53, 124.17, 135.64, 141.10, 168.40, 189.79, 189.88
13C Calc B	23.32, 28.85, 32.85, 42.71, 43.98, 57.16, 61.50, 62.56, 118.07, 118.50, 124.77, 135.67, 142.37, 168.52, 190.75, 190.78
1H Expt A	1.40, 2.01, 2.08, 2.50, 2.68, 3.09, 3.09, 3.09, 3.80, 6.79, 6.84, 6.93, 7.36
1H Expt B	1.32, 2.18, 2.22, 2.59, 2.77, 2.89, 2.89, 3.04, 3.80, 6.84, 6.89, 6.92, 7.36
1H Calc A	1.26, 2.02, 2.13, 2.22, 2.52, 3.00, 3.10, 3.12, 3.88, 7.14, 7.15, 7.25, 7.70
1H Calc B	1.20, 2.17, 2.19, 2.58, 2.79, 2.85, 2.89, 2.95, 3.88, 7.11, 7.21, 7.24, 7.70

CP3 thinks ExpA goes with CalcA and ExpB with CalcB:

ExpA with CalcA and ExpB with CalcB: 100,0%
ExpA with CalcB and ExpB with CalcA: 0,0%

CP3 values:

	C data	H data	All data
ExpA-CalcA & ExpB-CalcB	0,68	0,18	0,43
ExpA-CalcB & ExpB-CalcA	-1,44	-0,18	-0,81

Probabilities:

	C data	H data	All data
ExpA-CalcA & ExpB-CalcB	100,0%	88,1%	100,0%
ExpA-CalcB & ExpB-CalcA	0,0%	11,9%	0,0%

Figure S24. CP3 analysis of ^{1}H and ^{13}C NMR data of compound **5a** (**^{13}C NMR Expt A, ^{1}H NMR Expt A**) and **5b** (**^{13}C NMR Expt B, ^{1}H NMR Expt B**) before assignment of the signals and without linear regression. **^{1}H and ^{13}C NMR calc A** refer to **5a** candidate structure while **^{1}H and ^{13}C NMR calc B** refer to **5b** candidate structure.

1, 6, 2, 2a, 5a, 7’, 1A, 6a, 2’, 4’, 6’, 5’, 1’, 3’, C=O, C=O

Expt 5a

21.79(1), 23.83(6), 28.75(2), 35.27(2a), 36.89(5a), 55.03(7′), 55.43(1A), 56.08(6a), 112.23(2′), 114.52(4′), 118.62(6′), 129.87(5′), 132.71(1′), 160.09(3′), 178.54(C=O), 178.62(C=O)

Expt 5b

21.89(1), 23.56(6), 27.96(2), 35.31(2a), 36.68(5a), 55.42(7′), 56.68(1A), 57.61(6a), 112.40(2′), 114.57(4′), 119.03(6′), 129.78(5′), 133.76(1´), 160.09(3´), 179.60(C=O), 179.70(C=O)

Calc 5a

23.15(1), 29.82(6), 34.41(2), 42.48(2a), 43.82(5a), 57.21(7´), 59.02(1A), 60.28(6a), 117.97(2´), 118.53(4´), 124.17(6´), 135.64(5´), 141.10(1´), 168.40(3´), 189.79(C=O), 189.88(C=O)

Calc. 5b

23.32(1), 28.85(6), 32.85(2), 42.71(2a), 43.98(5a), 57.16(7´), 61.50(1A), 62.56(6a), 118.07(2´), 118.50(4´), 124.77(6´), 135.67(5´), 142.37(1´), 168.52(3´), 190.75(C=O), 190.78(C=O)

1, 2y, 6y, 2x, 6x, 2a, 5a, 6a, 7’, 2’, 6’, 4’, 5’

Expt 5a

1.40(1), 2.01(2y), 2.08(6y), 2.50(2x), 2.68(6x), 3.06(2a), 3.13(5a), 3.14(6a), 3.80(7´), 6.79(2´), 6.84(6´), 6.93(4´), 7.36(5´)

Expt 5b

1.32(1), 2.18(2y), 2.22(6y), 2.59(2x), 2.77(6x), 2.89(2a), 2.89(5a), 3.04(6a), 3.80(7´), 6.84(2´), 6.89(6´), 6.92(4´), 7.36(5´)

Calc. 5a

1.26(1), 2.02(2y), 2.13(6y), 2.22(2x), 2.52(6x), 3.00(2a), 3.10(5a), 3.12(6a), 3.88(7´), 7.14(2´), 7.15(6´), 7.25(4´), 7.70(5´)

Calc. 5b

1.20(1), 2.17(2y), 2.19(6y), 2.58(2x), 2.79(6x), 2.85(2a), 2.89(5a), 2.95(6a), 3.88(7´), 7.11(2´), 7.21(6´), 7.24(4´), 7.70(5´)

Enter two 1H and 13C experimental spectra then two calculated spectra:

13C Expt A	21.79(1), 23.83(6), 28.75(2), 35.27(2a), 36.89(5a), 55.03(7′), 55.43(1A), 56.08(6a), 112.23(2′), 114.52(4′), 118.62(6′), 129.87(5′), 132.71(1′), 160.09(3′), 178.54(C=O), 178.62(C=O)
13C Expt B	21.89(1), 23.56(6), 27.96(2), 35.31(2a), 36.68(5a), 55.42(7′), 56.68(1A), 57.61(6a), 112.40(2′), 114.57(4′), 119.03(6′), 129.78(5′), 133.76(1′), 160.09(3′), 179.60(C=O), 179.70(C=O)
13C Calc A	23.15(1), 29.82(6), 34.41(2), 42.48(2a), 43.82(5a), 57.21(7′), 59.02(1A), 60.28(6a), 117.97(2′), 118.53(4′), 124.17(6′), 135.64(5′), 141.10(1′), 168.40(3′), 189.79(C=O), 189.88(C=O)
13C Calc B	23.32(1), 28.85(6), 32.85(2), 42.71(2a), 43.98(5a), 57.16(7′), 61.50(1A), 62.56(6a), 118.07(2′), 118.50(4′), 124.77(6′), 135.67(5′), 142.37(1′), 168.52(3′), 190.75(C=O), 190.78(C=O)
1H Expt A	1.40(1), 2.01(2y), 2.08(6y), 2.50(2x), 2.68(6x), 3.06(2a), 3.13(5a), 3.14(6a), 3.80(7′), 6.79(2′), 6.84(6′), 6.93(4′), 7.36(5′)
1H Expt B	1.32(1), 2.18(2y), 2.22(6y), 2.59(2x), 2.77(6x), 2.89(2a), 2.89(5a), 3.04(6a), 3.80(7′), 6.84(2′), 6.89(6′), 6.92(4′), 7.36(5′)
1H Calc A	1.26(1), 2.02(2y), 2.13(6y), 2.22(2x), 2.52(6x), 3.00(2a), 3.10(5a), 3.12(6a), 3.88(7′), 7.14(2′), 7.15(6′), 7.25(4′), 7.70(5′)
1H Calc B	1.20(1), 2.17(2y), 2.19(6y), 2.58(2x), 2.79(6x), 2.85(2a), 2.89(5a), 2.95(6a), 3.88(7′), 7.11(2′), 7.21(6′), 7.24(4′), 7.70(5′)

```
CP3 thinks ExpA goes with CalcA and ExpB with CalcB:

 ExpA with CalcA and ExpB with CalcB: 100,0%
 ExpA with CalcB and ExpB with CalcA: 0,0%


CP3 values:
                            C data   H data   All data
 ExpA-CalcA & ExpB-CalcB     0,68     0,18      0,43
 ExpA-CalcB & ExpB-CalcA    -1,44    -0,18     -0,81


Probabilities:
                            C data   H data   All data
 ExpA-CalcA & ExpB-CalcB   100,0%    88,1%    100,0%
 ExpA-CalcB & ExpB-CalcA     0,0%    11,9%      0,0%
```

Figure S25. CP3 analysis of ^{1}H and ^{13}C NMR data of compound **5a (**^{13}**C NMR Expt A,** ^{1}**H NMR Expt A)** and **5b** (^{13}**C NMR Expt B,** ^{1}**H NMR Expt B**) before assignment of the signals and without linear regression. ^{1}**H and** ^{13}**C NMR calc A** refer to **5a** candidate structure while ^{1}**H and** ^{13}**C NMR calc B** refer to **5b** candidate structure.
